# A Type-2 Block-Component-Decomposition Based 2D AOA Estimation Algorithm for an Electromagnetic Vector Sensor Array

**DOI:** 10.3390/s17050963

**Published:** 2017-04-27

**Authors:** Yu-Fei Gao, Guan Gui, Wei Xie, Yan-Bin Zou, Yue Yang, Qun Wan

**Affiliations:** 1 School of Electronic Engineering, University of Electronic Science and Technology of China, No. 2006, Xiyuan Ave., West Hi-Tech Zone, Chengdu 611731, China; yufeiee@gmail.com (Y.-F.G.); raysheik@foxmail.com (W.X.); 201311020327@std.uestc.edu.cn (Y.-B.Z.); 201611020223@std.uestc.edu.cn (Y.Y.); wanqun@uestc.edu.cn (Q.W.); 2 College of Telecommunication and Information Engineering, Nanjing University of Posts and Telecommunications, 66 Xinmofan Road, Nanjing 210003, China

**Keywords:** EMVS, L-shaped array, partial polarization, 2D AOA, rank-(*L*_1_, *L*_2_,·) BCD, tensor decomposition

## Abstract

This paper investigates a two-dimensional angle of arrival (2D AOA) estimation algorithm for the electromagnetic vector sensor (EMVS) array based on Type-2 block component decomposition (BCD) tensor modeling. Such a tensor decomposition method can take full advantage of the multidimensional structural information of electromagnetic signals to accomplish blind estimation for array parameters with higher resolution. However, existing tensor decomposition methods encounter many restrictions in applications of the EMVS array, such as the strict requirement for uniqueness conditions of decomposition, the inability to handle partially-polarized signals, etc. To solve these problems, this paper investigates tensor modeling for partially-polarized signals of an L-shaped EMVS array. The 2D AOA estimation algorithm based on rank-$\left(L_{1},L_{2},\cdot \right)$ BCD is developed, and the uniqueness condition of decomposition is analyzed. By means of the estimated steering matrix, the proposed algorithm can automatically achieve angle pair-matching. Numerical experiments demonstrate that the present algorithm has the advantages of both accuracy and robustness of parameter estimation. Even under the conditions of lower SNR, small angular separation and limited snapshots, the proposed algorithm still possesses better performance than subspace methods and the canonical polyadic decomposition (CPD) method.

## 1. Introduction

Array signal processing is an important branch of the information acquisition and detection field, which has been studied and applied extensively in the academic and industrial communities for nearly half a century [1,2,3]. The traditional array usually consists of scalar sensors, which are able to receive a one-dimensional signal only. However, electromagnetic waves have diverse magnetic and electric field components. How to make the best of the complete information of the electromagnetic wave has been an important subject that has been paid close attention by researchers. The EMVS is a kind of polarization-sensitive antenna, which can receive magnetic and electric field information of the signal simultaneously [4]. Compared with the traditional scalar sensor, EMVS can sense electromagnetic waves with different polarization forms and fully exploit information carried by the wave itself. Since the 1980s, many investigations have been presented to study the problem of parameter estimation in EMVS arrays. In 1981, R.T. Compton put forward an adaptive signal processing method of the tripole antenna array, which can obtain better anti-disturbance performance than traditional array processing methods [5]. In 1992, J. Li et al. introduced the rotational invariance techniques (ESPRIT) algorithm to the signal processing of the EMVS array to present a joint estimation algorithm of the AOA-polarization parameter [6]. In 1994, A. Nehorai and E. Paldi studied the received signal model of the EMVS array and CRB analysis of the parameter estimation comprehensively, in which the mean square angular error (MSAE) and the covariance vector angular error (CVAE) were introduced [7]. In 1996, K.C. Tan et al. analyzed the linear independence of steering vectors of EMVS for different polarization signal types [8]. In 2000, K.T. Wong and M.D. Zoltowski developed an algorithm of AOA and polarization parameter estimation with a closed-form solution for the EMVS array, by which high precision estimation could be achieved [9]. In 2004, D. Rahamim et al. proposed a preprocessing algorithm of coherence signals over the polarization domain, which can be applied to the EMVS array arbitrarily distributed in space [10]. In 2012, X. Yuan constructed a joint estimation algorithm of AOA-polarization parameters for the polynomial-phase signal with full polarization and derived the corresponding analytical expression of CRB [11]. It can be seen that EMVS array signal processing has been established as a relatively complete theoretical system through the past 30 years.

All of the methods mentioned above fall into one-dimensional vector or two-dimensional matrix modeling. However, received signals of the EMVS array have a multidimensional structure naturally, and the structural information associated with parameters exists among each dimension [12,13,14,15]. The traditional methods stretch the multidimensional data into matrices or vectors, which may lead to the loss of the structural information. In recent years, tensor-decomposition-based methods are more and more applied in the signal processing field [16,17,18]. Compared with matrix and vector modeling, the tensor modeling is more suitable to the real signal with a multidimensional structure. Besides, tensor decomposition possesses the blind estimation feature, which can directly estimate the source signal from measurements without the need for any prior information. With the help of such characteristics, even if the noise is non-Gaussian, tensor-based methods can still be applied to achieve parameter estimation. Tensor decomposition was proposed in psychometrics first in the 1970s [19], which also has been obtained by in-depth investigation in phonetics [20] and chemometrics [21]. In the 1990s, the higher order statistics (HOS) attracted attention in the signal processing field [22]. It is found that HOS is essentially interconnected with tensor modeling. In fact, the HOS-based independent component analysis (ICA) [23,24] implied the idea of tensor decomposition. Combining the tensor decomposition model with the array manifold, the canonical polyadic decomposition (CPD) method has been introduced into the parameter estimation of 2D array signal processing [25,26]. In 2005, S. Miron et al. introduced the tensor modeling to the EMVS array and proposed a tensor-based multiple signal classification (MUSIC) algorithm [12]. However, this search algorithm only considered the invariances in the polarization dimension, i.e., only using the uni-mode orthogonality. In 2009, X.F. Gong et al. put forward a two-fold mode-projection MUSIC (TM-MUSIC) algorithm, applying it to the EMVS array [27], which can obtain a better performance than the long-vector MUSIC algorithm by fusing the orthogonality on both dimensions and taking full advantage of the structural information of the tensor data. In 2011, X.J. Guo et al. applied the CPD algorithm to the EMVS array and derived the uniqueness conditions of decomposition, as well as the identifiability of AOAs for coherent signals [28]. In 2014, K. Han and A. Nehorai constructed a nested vector sensor array and introduced the tensor modeling for AOA estimation [29]. In 2015, X.R. Zhang et al. presented an adaptive beamformer for the EMVS array based on tensor modeling with a two-stage technique [30], which has a good robustness even under the condition of steering vector mismatching. In 2016, P. Forster et al. analyzed the theoretical performance of the tensor MUSIC and classic long vector MUSIC in polarized source estimation and gave the corresponding theoretical mean square error (MSE) [31].

The existing tensor-decomposition-based parameter estimation methods for the EMVS array are mostly based on the CPD modeling. Such a model has the advantage of uniqueness, but decomposition factors must be rank-1, which may not be satisfied in practice [32]. Therefore, the signal processing community hopes to find a new framework that not only can maintain the uniqueness of decomposition, but also that does not need the severe requirement for rank constraints. In 2008, L. De Lathauwer proposed a tensor decomposition model combined with the features of CPD and Tucker decomposition (TKD), called the block component decomposition (BCD), or the block term decompositions (BTD) [33,34,35]. This model has various forms of decomposition. For instance, a rank-$\left(L,L,1\right)$ BCD expresses the factors as the outer product of one rank-*L* matrix with a vector. Such a model has been effectively applied in blind source separation [36,37], in which a rank-$\left(2,2,1\right)$ BCD model is utilized to match data with limited samples. When the reflections result in the inter-symbol-interference (ISI) occurring simultaneously on both the near and far field, a more flexible block-CPD model can be applied [38]. This shows that the BCD model has more flexible applicability than the CPD. Another one is rank-$\left(L_{1},L_{2},\cdot \right)$ BCD, which expresses the factors as the product of a core tensor with two factor matrices. In 2008, D. Nion and L. De Lathauwer introduced such a model into the DS-CDMA (Direct Sequence-Code Division Multiple Access) system to construct a blind receiver used for convolutive mixtures. It can achieve a good performance on both channel identification and symbol estimation [39]. In 2013, combining the partial least squares (PLS) with BCD modeling, Q.B. Zhao et al. put forward a higher order PLS method that can achieve good results in 3D movement trajectories decoding for the electrocorticogram (ECoG) signal [40]. In 2014, B. Hunyadi et al. introduced the BCD into electroencephalogram signal processing [41], in which a better performance is gained in the ictal pattern identification than the CPD algorithm. In addition, these types of BCD models also include the PARALIND (PARAllel factors with LINear Dependency) [42,43], which has mainly been utilized in blind beamforming and multi-antenna coding [44]. In 2012, M. Sorensen and L. De Lathauwer proposed a rank-$\left(L_{1},L_{2},\cdot \right)$ BCD algorithm with Vandermonde factors, which has good estimation performance in the uniform linear array (ULA) [45]. In 2013, L. Sorber et al. generalized the CPD and rank-$\left(L_{r},L_{r},1\right)$ BCD method to propose a optimization-based tensor decomposition algorithm that can diminish computation source requirement by means of Jacobian’s Gramian structure [46]. In 2015, P. Tichavsky et al. proposed a non-orthogonal tensor diagonalization BCD algorithm, which has a nearly quadratic convergence performance [47]. In 2016, X.F. Gong et al. put forward two kinds of coupled block simultaneous generalized Schur decomposition (SGSD)-based BCD algorithms, which have advantages of low computational resource requirement and high computation accuracy [48]. So far, there has been little public research on the application of the BCD in the vector sensor array. As discussed above, the received signal model of the EMVS array has a natural multidimensional structure. Additionally, for 2D AOA, the angle pair-matching is a troublesome problem. The earlier research works often need complicated searching methods to achieve pair-matching [49,50,51,52]. Some researchers put forward the automatic pairing method by using the eigenvalue of the transfer matrix, but the issue of eigenvalue ordering has not been well solved with such an algorithm [53]. Since the BCD method possesses the blind estimation feature, it can solve such a problem properly. This paper takes this as the research motivation to investigate the parameter estimation algorithm based on the BCD modeling for the EMVS array.

The key contribution of this paper is the rank-$\left(L_{1},L_{2},\cdot \right)$ BCD modeling of the partial polarization signal incident on an EMVS array. For achieving the AOA parameters estimation, an iterative algorithm is proposed to compute each factor of such a BCD model. This algorithm adopts a block diagonal constraint criterion of the steering matrix and constructs a subspace-like estimator to achieve the estimation of spatial frequencies. The BCD-based methods have a common feature that the performance of the algorithm can be guaranteed only if the rank conditions are satisfied. Thus, we analyze whether or not the rank conditions can be met. Additionally, for the partial polarization signal, the decomposition uniqueness of rank-$\left(L_{1},L_{2},\cdot \right)$ BCD is investigated in this paper. The proposed method can accomplish the pair-matching of azimuth and elevation angles automatically by means of the decomposition uniqueness of the BCD model.

The other sections of this paper are organized as follows: Section 2 describes the received signal model of the EMVS array and introduces the BCD method; Section 3 develops the algorithm based on rank-$\left(L_{1},L_{2},\cdot \right)$ BCD for 2D AOA estimation; Section 4 presents numerical simulations for verifying the proposed algorithm and demonstrates the comparison with the existing methods; finally, the last section concludes this paper.

## 2. Data Model

For convenience of statement hereafter, several notions are explained as follows. The scalar is indicated by italic letters, e.g., *x*. The vector and matrix are indicated by bold italic lower- and upper-case letters, e.g., x,X. The tensor is indicated by calligraphic capital letter, e.g., X. ${∥X∥}_{F}$ is the Frobenius norm of the tensor X, expressed as the square root of the sum of the absolute squares of its elements. The operator ⊗ denotes the Kronecker product between matrices. The operator ⊙ indicates the column-wise Kronecker product, or the Khatri–Rao product [16]. The operator $\times_{n}$ denotes the mode-*n* product between the tensor and matrix, where the mode is considered as the order of the tensor data. For a third-order tensor $X\in C^{M\times N\times P}$, $X_{\left(1\right)}$ is defined as the the unfolded matrix, or matricization of X in mode-1, i.e., $X_{\left(1\right)}=\left[X_{1},X_{2},\ldots ,X_{P}\right]\in C^{M\times \left(PN\right)}$; similarly, $X_{\left(2\right)}$ and $X_{\left(3\right)}$ denote the matricization in mode-2 and mode-3, respectively. The mode-*n* rank of tensor X is the rank of its unfolded matrix $X_{\left(n\right)}$.

### 2.1. EMVS Array Signal Model

**Figure 1:**
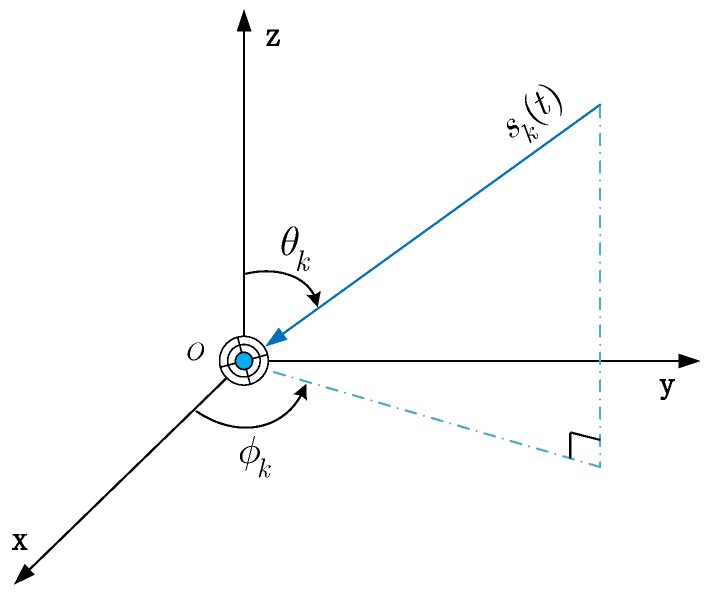
The geometry of a single EMVS.

Under the assumption of narrowband and far-field signal [7,8], the measurement of EMVS can be expressed as:

(1) $$\begin{array}{cc} y_{k}\left(t\right) & =\Theta_{k}\xi_{k}\left(t\right)\\ & =\left[\begin{array}{c} e_{x}\\ e_{y}\\ e_{z}\\ h_{x}\\ h_{y}\\ h_{z} \end{array}\right]=\left[\begin{array}{cc} -\sin\phi_{k} & -\cos\phi_{k}\cos\theta_{k}\\ \cos\phi_{k} & -\sin\phi_{k}\cos\theta_{k}\\ 0 & \sin\theta_{k}\\ -\cos\phi_{k}\cos\theta_{k} & \sin\phi_{k}\\ -\sin\phi_{k}\cos\theta_{k} & -\cos\phi_{k}\\ \sin\theta_{k} & 0 \end{array}\right]\xi_{k}\left(t\right), \end{array}$$

where $\phi_{k},\theta_{k}$ denote the azimuth and elevation angle of arrival signal, respectively, as shown in Figure 1.

**Figure 2:**
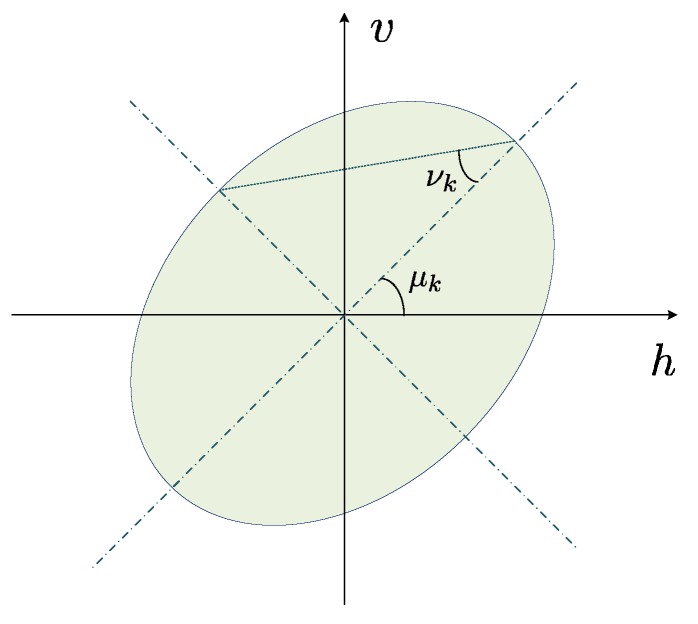
The electromagnetic polarization ellipse.

For a fully-polarized signal, $\xi_{k}\left(t\right)=Q_{k}w_{k}s_{k}\left(t\right)=g_{k}s_{k}\left(t\right)$, in which $s_{k}\left(t\right)$ is the *k*-th source signal, $Q_{k}$ and $w_{k}$ are the variables associated to polarization parameters.

(2) $$Q_{k}=\left[\begin{array}{cc} \cos\mu_{k} & \sin\mu_{k}\\ -\sin\mu_{k} & \cos\mu_{k} \end{array}\right],\;w_{k}=\left[\begin{array}{c} \cos\nu_{k}\\ j\sin\nu_{k} \end{array}\right],$$

where $\mu_{k}\in \left[-\pi /2,\pi /2\right]$ is the polarization orientation angle of the electromagnetic wave and $\nu_{k}\in \left[-\pi /4,\pi /4\right]$ is the ellipticity angle, as shown in Figure 2. Thus, $y_{k}\left(t\right)=\Theta_{k}g_{k}s_{k}\left(t\right)=b_{k}s_{k}\left(t\right)$, where $b_{k}\in C^{6\times 1}$ is referred to as the polarization steering vector corresponding to the *k*-th signal.

**Figure 3:**
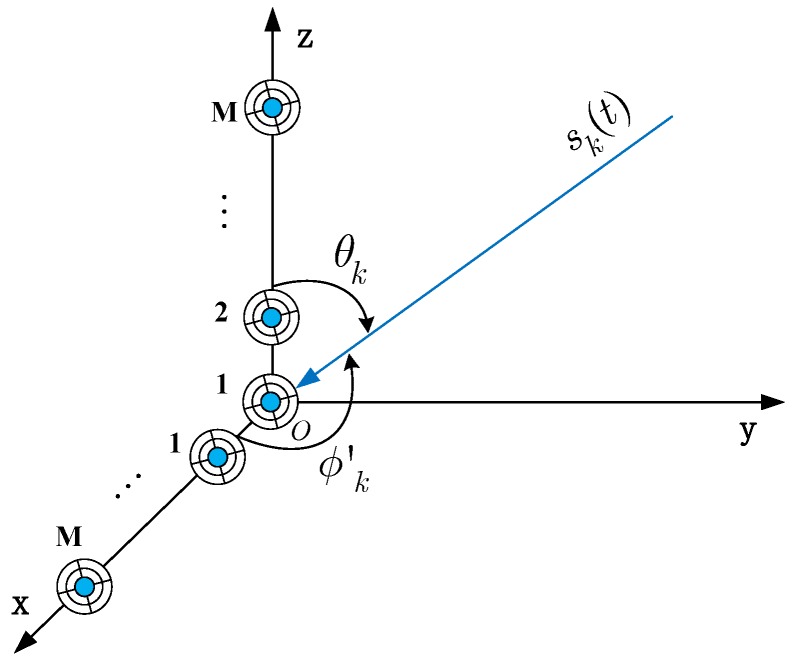
L-shaped array configuration of the EMVS array.

The above signal model is only for single EMVS at the receiver. For an EMVS array, the manifold configuration of sensors must be dealt with. This paper considers the L-shaped array [54,55] shown in Figure 3, which consists of a couple of orthogonal uniform linear arrays, and each of them has *M* sensors. Given the polarization steering matrix as $B=\left[b_{1},\ldots ,b_{K}\right]\in C^{6\times K}$ and considering noise, the received signal model of the EMVS array can be expressed as:

(3) $$\begin{array}{cc} x\left(t\right) & =\left(A_{x}⊙B\right)s^{T}\left(t\right)+n_{x}\left(t\right), \end{array}$$

(4) $$\begin{array}{cc} z\left(t\right) & =\left(A_{z}⊙B\right)s^{T}\left(t\right)+n_{z}\left(t\right), \end{array}$$

where $A_{x}=\left[a_{x,1},a_{x,2},\ldots ,a_{x,K}\right]\in C^{M\times K}$ is the steering matrix of the subarray along the *x*-axis. Without loss of generality, assuming the array element spatial interval as *d* and letting $\alpha_{k}=-\frac{2\pi d}{\lambda }\cos\phi_{k}^{\prime }$, in which λ is the wavelength of source signal, we have $a_{x,k}={\left[1,e^{j\alpha_{k}},\cdots ,e^{j\left(M-1\right)\alpha_{k}}\right]}^{T}$. $s\left(t\right)\in C^{1\times K}$ is the source signal, vector and $n_{x}\left(t\right)\in C^{M\times 1}$ is the additive white Gaussian noise (AWGN) vector. The signal model of the subarray along the *z*-axis is consistent with the form of the *x*-axis.

In practical applications, the fully-polarized signal appears rarely. Most signals are partially polarized. The trajectory of the endpoint of the electric field vector at the spatial frequency is no longer a certain ellipse, but a curve of shape and direction that changes over time. In this case, the polarization parameters are not constant. Thus, $g_{k}$ cannot be incorporated into the polarization steering vector $b_{k}$. For instance, the circularly-polarized signal has $\xi_{k}\left(t\right)=Q_{k}\left[w{\tilde{s}}_{1}^{\left(k\right)}\left(t\right)+w^{*}{\tilde{s}}_{2}^{\left(k\right)}\left(t\right)\right]$, where $w=\sqrt{2}/2{\left[1,j\right]}^{T}$ and $\left({\tilde{s}}_{1}^{\left(k\right)}\left(t\right),{\tilde{s}}_{2}^{\left(k\right)}\left(t\right)\right)$ are the *k*-th signal complex envelope [8]. For such a partially-polarized signal, the received signal model of the EMVS array can be rewritten as:

(5) $$\begin{array}{cc} x\left(t\right) & =\left(A_{x}{⊙}_{b}\Psi \right)s^{T}\left(t\right)+n_{x}\left(t\right), \end{array}$$

(6) $$\begin{array}{cc} z\left(t\right) & =\left(A_{z}{⊙}_{b}\Psi \right)s^{T}\left(t\right)+n_{z}\left(t\right), \end{array}$$

where ${⊙}_{b}$ denotes the Khatri–Rao product with block form [33]; $\Psi =\left[\Theta_{1},\ldots ,\Theta_{K}\right]\in R^{6\times 2K}$; $s\left(t\right)=\left[{\tilde{s}}_{1}^{\left(1\right)}\left(t\right),{\tilde{s}}_{2}^{\left(1\right)}\left(t\right),\ldots ,{\tilde{s}}_{1}^{\left(K\right)}\left(t\right),{\tilde{s}}_{2}^{\left(K\right)}\left(t\right)\right]\in C^{1\times 2K}$ is the vector of partially-polarized signals. The signal model of the subarray along the *z*-axis has the same form as that along the *x*-axis. Without ambiguity, the argument *t* is omitted in the following discussions for simplicity.

For *N* snapshots, the received signal models (5) and (6) become:

(7) $$\begin{array}{cc} X & =\left(A_{x}{⊙}_{b}\Psi \right)S^{T}+N_{x}, \end{array}$$

(8) $$\begin{array}{cc} Z & =\left(A_{z}{⊙}_{b}\Psi \right)S^{T}+N_{z}, \end{array}$$

where $S\in C^{N\times 2K}$ is the source signal matrix for the *N* snapshots and $N_{x},N_{z}\in C^{M\times N}$ are the matrices of AWGN on the subarrays along the *x*- and *z*-axis, respectively.

### 2.2. Block Component Decomposition

As the combination of CPD and TKD, the BCD framework maintains the advantages of both, the former with decomposition uniqueness and the latter with the relaxing rank requirement for the factors [33,34,35]. In addition, BCD has the feature of structural diversity, such as rank-$\left(L,L,1\right)$, rank-$\left(L_{1},L_{2},L_{3}\right)$ and rank-$\left(L_{1},L_{2},\cdot \right)$ patterns. Different patterns have different forms of decomposition, corresponding to different signal models. This paper mainly pays attention to the rank-$\left(L_{1},L_{2},\cdot \right)$ BCD, i.e., the Type-2 BCD model.

**Definition** **1.** *Type-2 BCD in rank-*$\left(L_{1},L_{2},\cdot \right)$ *terms:*

*The decomposition of a tensor* $X\in C^{M\times P\times N}$ *is called the Type-2 block component decomposition, if it can be expressed as the summation of a series rank-*$\left(L_{1},L_{2},\cdot \right)$ *factors,*

(9) $$X=\sum_{k=1}^{K}X_{k}=\sum_{k=1}^{K}C_{k}\times_{1}A_{k}\times_{2}B_{k},$$

**Figure 4:**
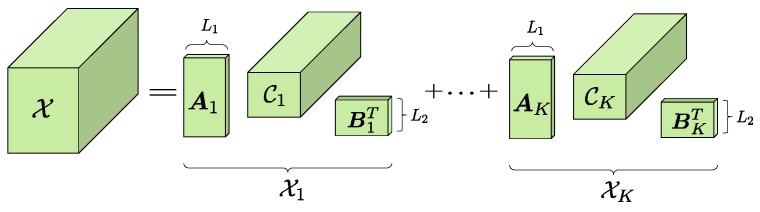
Type-2 BCD in rank-$\left(L_{1},L_{2},\cdot \right)$ terms.

*where* $C_{k}\in C^{L_{1}\times L_{2}\times N}$ *can be termed as the core tensor of the k-th component. The mode-1 rank and mode-2 rank of* $C_{k}$ *are* $L_{1}$ *and* $L_{2}$*, respectively. Both* $A_{k}\in C^{M\times L_{1}}$ *and* $B_{k}\in C^{P\times L_{2}}$ *are of full column rank. The type-2 BCD model in rank-*$\left(L_{1},L_{2},\cdot \right)$ *terms is schematized in Figure 4.*

The BCD model can also be expressed as:

(10) $$X=C\times_{1}A\times_{2}B,$$

where $A=\left[A_{1},\ldots ,A_{K}\right],B=\left[B_{1},\ldots ,B_{K}\right]$, and $C\in C^{KL_{1}\times KL_{2}\times N}$ is defined as:

(11) $${\left[C\right]}_{\left(k-1\right)L_{1}+l_{1},\left(k-1\right)L_{2}+l_{2},n}={\left[C_{k}\right]}_{l_{1},l_{2},n},\;\forall l_{1},l_{2},n,k,$$

and Equation (10) can be written as X=⟦A,B,C⟧ (cf. [16]).

## 3. The Proposed Method

### 3.1. The BCD Modeling of the EMVS Array

Consider the observation data on both subarrays [8]; we have the received signal model,

(12) $$y=\sum_{k=1}^{K}A_{k}⊗\Theta_{k}s_{k}^{T}+n.$$

The corresponding matrix form is:

(13) $$Y=\left(A{⊙}_{b}\Psi \right)S^{T}+N,$$

in which $A=\left[A_{1},\ldots ,A_{K}\right]$, $A_{k}=blockdiag\left(a_{x,k},a_{z,k}\right)$, $\Psi =\left[\Theta_{1},\ldots ,\Theta_{K}\right]$, $S=\left[S_{1}^{T},\ldots ,S_{K}^{T}\right]$ and $S_{k}=\left[s_{k}{\left(1\right)}^{T},\ldots ,s_{k}{\left(N\right)}^{T}\right]$, where $blockdiag\left(\cdot \right)$ denotes a block diagonal matrix. Note that such a model is the rank-$\left(L_{1},L_{2},\cdot \right)$ BCD [34], where $L_{1}=L_{2}=2$. Then, Equation (13) can be rewritten as:

(14) $$Y=\sum_{k=1}^{K}S_{k}\times_{1}A_{k}\times_{2}\Theta_{k}+N,$$

where $Y\in C^{\left(ML_{1}\right)\times 6\times N}$ is the received signal; $S_{k}\in C^{L_{1}\times L_{2}\times N}$ is the source signal, of which the mode-3 matricization is $S_{k}^{T}$, i.e., $S_{k}^{T}={\left[S_{k}\right]}_{\left(3\right)}$. N is the corresponding AWGN.

Next, we investigate if the rank condition of each factor is satisfied in Equation (14). First, the steering matrix ${\left\{A_{k}\right\}}_{k=1}^{K}\in C^{ML_{1}\times L_{1}}$ is of full column rank when M≥1. The same is true for ${\left\{\Theta_{k}\right\}}_{k=1}^{K}\in R^{6\times L_{2}}$ when $L_{2}<6$. For ${\left\{S_{k}\right\}}_{k=1}^{K}\in C^{L_{1}\times L_{2}\times N}$, the size of mode-1 matricization is $\left(L_{1}\times NL_{2}\right)$ and $\left(L_{2}\times NL_{1}\right)$ for mode-2. Thus, the mode-1 and mode-2 rank of ${\left\{S_{k}\right\}}_{k=1}^{K}$ are $L_{1}$ and $L_{2}$, respectively, when snapshot *N* is sufficiently large. Consequently, the rank conditions of each decomposition factor in Equation (14) can be satisfied if:

(15) $$\left\{\begin{array}{c} M\geq 1,\\ L_{2}\leq 6,\\ N\geq \max\left(\left\lceil \frac{L_{1}}{L_{2}}\right\rceil ,\left\lceil \frac{L_{2}}{L_{1}}\right\rceil \right). \end{array}\right.$$

For the L-shaped array in this paper, on each subarray, the number of sensors M>1 must be true. Additionally, since $L_{1}=L_{2}=2$, no matter what the snapshots are, multiple or single, Equation (14) will be valid according to Equation (15).

#### 3.1.1. Uniqueness Analysis

Based on the EMVS array signal model, the decomposition uniqueness of Equation (14) is investigated in this section.

**Definition** **2.** *Essentially unique for rank-*$\left(L_{1},L_{2},\cdot \right)$ *BCD:*

*The rank-*$\left(L_{1},L_{2},\cdot \right)$ *BCD described in Equation (14) is called essentially unique, if the factors satisfy the following relationships: (1) each of* $A_{k}$ *and* $\Theta_{k}$ *can be post-multiplied by a nonsingular matrix, i.e.,* ${\bar{A}}_{k}=A_{k}\Gamma_{k},{\bar{\Theta }}_{k}=\Theta_{k}\Delta_{k}$*, in which* $\Gamma_{k}\in C^{L_{1}\times L_{1}},\Delta_{k}\in C^{L_{2}\times L_{2}}$*; (2) corresponding to Condition 1, there exists the transformation of* $S_{k}$: ${\bar{S}}_{k}=S_{k}\times_{1}\Gamma_{k}^{-1}\times_{2}\Delta_{k}^{-1}$*.*

If $\bar{A}=\left[{\bar{A}}_{1},\ldots ,{\bar{A}}_{K}\right]$, $\bar{\Psi }=\left[{\bar{\Theta }}_{1},\ldots ,{\bar{\Theta }}_{K}\right]$ and ${\left[\bar{S}\right]}_{\left(k-1\right)L_{1}+l_{1},\left(k-1\right)L_{2}+l_{2},n}={\left[{\bar{S}}_{k}\right]}_{l_{1},l_{2},n},\;\forall l_{1},l_{2},n,k$, the two sets of BCD expressions $〚\bar{A},\bar{\Psi },\bar{S}〛$ and $〚A,\Psi ,S〛$ can be considered as essentially unique.

**Theorem** **1.** Uniqueness of BCD modeling for the EMVS array:

*The rank-*$\left(L_{1},L_{2},\cdot \right)$ *BCD descried in Equation (14) is essentially unique, if* A *and* **Ψ** *are of full column rank,* N≥3 *and all elements of* ${\left\{S_{k}\right\}}_{k=1}^{K}$ *are characterized by jointly continuous probability density functions.*

For the proof of Theorem 1, reference can be made to [34]. Since the EMVS model in this paper is based on the assumption that source signals obey the zero mean Gaussian random process [7], this implies that the elements of ${\left\{S_{k}\right\}}_{k=1}^{K}$ are surely characterized by jointly continuous probability density functions, i.e., the conditions of factors ${\left\{S_{k}\right\}}_{k=1}^{K}$ in Equation (14) are satisfied from Theorem 1. In addition, the array manifold based on the L-shaped array in this paper gives $L_{1}=L_{2}=2$; the structure of ${\left\{A_{k}\right\}}_{k=1}^{K}$ is block diagonal; and each block factor is the steering vector of the subarray, which has the Vandermonde structure. As a consequence, the arbitrary two columns of A are linearly independent. Hence, $rank\left(A\right)=\min\left(2M,2K\right)$. Thus, when M>K, A is of full column rank. From the definition of ${\left\{\Theta_{k}\right\}}_{k=1}^{K}$ in Section 2.1, we have $rank\left(\Psi \right)=\min\left(6,2K\right)$. Thus, when K<3, Ψ is of full column rank. The above analysis reveals that the decomposition from the tensor model (14) of the EMVS array is unique, if the following conditions are met,

(16) $$\left\{\begin{array}{c} K\leq \min\left(M,3\right),\\ N\geq 3. \end{array}\right.$$

It should be noted that Equation (16) is only the sufficient condition. In practical applications, if the constraints are imposed on factor matrices, the decomposition (14) can still be unique, even if the conditions limited by Equation (16) may not be satisfied.

### 3.2. AOA Estimation Algorithm

Given the rank-$\left(L_{1},L_{2},\cdot \right)$ BCD model (14) with additive noise, the task is to acquire the estimation of factor matrices ${\left\{A_{k}\right\}}_{k=1}^{K}$, ${\left\{\Theta_{k}\right\}}_{k=1}^{K}$ from the received signal $Y\in C^{\left(2M\right)\times 6\times N}$ and obtain the estimation of spatial frequency ${\left\{\alpha_{k}\right\}}_{k=1}^{K}$. This paper adopts the minimum mean square error (MMSE) criterion [35],

(17) $$\begin{array}{c} \begin{array}{cc} & \min_{{\hat{A}}_{k},{\hat{\Theta }}_{k}}{∥Y-\hat{Y}∥}_{F}^{2},\;k=1,2,\ldots ,K,\\ s.t.\; & \hat{Y}=\sum_{k=1}^{K}{\hat{S}}_{k}\times_{1}{\hat{A}}_{k}\times_{2}{\hat{\Theta }}_{k}. \end{array} \end{array}$$

With the mode-*n* matricizations of the tensor Y and N, i.e., $Y_{\left(n\right)}$ and $N_{\left(n\right)}$, n=1,2,3, the signal model (14) can be rewritten as:

(18) $$\begin{array}{cc} Y_{\left(1\right)} & =A{\left[F_{1}^{T},F_{2}^{T},\ldots ,F_{K}^{T}\right]}^{T}+N_{\left(1\right)}, \end{array}$$

(19) $$\begin{array}{cc} Y_{\left(2\right)} & =\Psi {\left[G_{1}^{T},G_{2}^{T},\ldots ,G_{K}^{T}\right]}^{T}+N_{\left(2\right)}, \end{array}$$

(20) $$\begin{array}{cc} Y_{\left(3\right)} & =S{\left(A{⊙}_{b}\Psi \right)}^{T}+N_{\left(3\right)}, \end{array}$$

in which $F_{k}={\left[S_{k}\times_{2}\Theta_{k}\right]}_{\left(1\right)}$, $G_{k}={\left[S_{k}\times_{1}A_{k}\right]}_{\left(2\right)}$, k=1,2,…,K.

Several algorithms can be employed to solve the above problems [35,46,56,57]. The typical one is alternating least squares (ALS) [19,46]. With two fixed components of A,Ψ,S in each iteration, the remaining one is updated by the least squares approach. Then, the same scheme is repeated in turn until the convergence condition is satisfied.

For instance, with ${\left\{S_{k}\right\}}_{k=1}^{K}$ and ${\left\{\Theta_{k}\right\}}_{k=1}^{K}$ fixed, the update of A is calculated as follows,

(21) $$\min_{\hat{A}}{∥Y_{\left(1\right)}-\hat{A}{\left[F_{1}^{T},F_{2}^{T},\ldots ,F_{K}^{T}\right]}^{T}∥}_{F}^{2},$$

then we can obtain:

(22) $$\hat{A}=Y_{\left(1\right)}{\left({\left[{\hat{F}}_{1}^{T},{\hat{F}}_{2}^{T},\ldots ,{\hat{F}}_{K}^{T}\right]}^{T}\right)}^{†},$$

where ${\left(\cdot \right)}^{†}$ represents the pseudo-inverse of a matrix. With ${\left\{S_{k}\right\}}_{k=1}^{K}$ and ${\left\{A_{k}\right\}}_{k=1}^{K}$ fixed, the same procedure gives the update of Ψ,

(23) $$\hat{\Psi }=Y_{\left(2\right)}{\left({\left[{\hat{G}}_{1}^{T},{\hat{G}}_{2}^{T},\ldots ,{\hat{G}}_{K}^{T}\right]}^{T}\right)}^{†}.$$

Then, fixing ${\left\{A_{k}\right\}}_{k=1}^{K}$ and ${\left\{\Theta_{k}\right\}}_{k=1}^{K}$ leads to the update of S,

(24) $$\hat{S}=Y_{\left(3\right)}{\left({\left[\hat{A}{⊙}_{b}\hat{\Psi }\right]}^{T}\right)}^{†}.$$

During the steps of updating A and Ψ, the normalization by using QR decomposition can be added to enhance the stability of estimation [35]. It is necessary to guarantee that $\left[{\hat{F}}_{1}^{T},{\hat{F}}_{2}^{T},\ldots ,{\hat{F}}_{K}^{T}\right]$, $\left[{\hat{G}}_{1}^{T},{\hat{G}}_{2}^{T},\ldots ,{\hat{G}}_{K}^{T}\right]$ and $\left(\hat{A}{⊙}_{b}\hat{\Psi }\right)$ are of full column rank in the iterations.

In addition, the structural information about factor matrices can be considered in the initialization step for accelerating the convergence of ALS. Let $Y_{n}$ represent the *n*-th frontal slice matrix of the tensor Y, i.e., $Y_{n}={\left[Y\right]}_{:,:,n},\;n=1,2,\ldots ,N$; then given $L_{1}=L_{2}$, we have:

(25) $$Y_{2}Y_{1}^{†}=A\cdot blockdiag\left({\ddot{S}}_{1},{\ddot{S}}_{2},\ldots ,{\ddot{S}}_{K}\right)\cdot A^{†},$$

where ${\ddot{S}}_{k}={\left[S_{k}\right]}_{:,:,2}\cdot {\left[S_{k}\right]}_{:,:,1}^{†}$ is of full rank, known from the previous rank condition of rank-$\left(L_{1},L_{2},\cdot \right)$ BCD and the characteristics of the source signal. A is also full column rank in terms of the uniqueness analysis because Equation (16) is fulfilled. This means that the column space of $Y_{2}Y_{1}^{†}$ is the same as that of A. A similar conclusion can be deduced that $Y_{2}^{T}{\left(Y_{1}^{T}\right)}^{†}$ and Ψ have the same column space. Therefore, we can employ singular value decomposition (SVD) to initiate the factor matrices for the ALS algorithm.

The analysis of Section 3.1 shows that solving Equation (14) can result in the estimation of $A_{k},\Theta_{k},k=1,\ldots K$. After finding the steering matrix ${\hat{A}}_{k}$ by means of ALS, the spacial frequency can be obtained. Note that the trivial indeterminacies of ${\hat{A}}_{k}$ exists, i.e.,

(26) $${\hat{A}}_{k}=A_{k}\Gamma_{k},$$

where $\Gamma_{k}\in C^{L_{1}\times L_{1}}$ is a nonsingular matrix. Equation (26) indicates that the BCD estimation result and steering matrix have the same column space. Because $A_{k}$ has the block diagonal structure, we can extract the correct AOA information by exploiting this feature.

For the array manifold configuration in this paper, the $A_{k}$ can be parted as $A_{k}={\left[A_{k,1}^{T},A_{k,2}^{T}\right]}^{T}$, where $A_{k,1}=\left[a_{x,k},0\right]$, $A_{k,2}=\left[0,a_{z,k}\right]$. In the same way, the estimation of ${\hat{A}}_{k}$ is parted as ${\hat{A}}_{k}={\left[{\hat{A}}_{k,1}^{T},{\hat{A}}_{k,2}^{T}\right]}^{T}$. Then, combined with Equation (26), we have:

(27) $$\begin{array}{cc} {\hat{A}}_{k,1} & =\left[a_{x,k},a_{x,k}\right]\cdot diag\left({\left[\Gamma_{k}\right]}_{1,:}\right), \end{array}$$

(28) $$\begin{array}{cc} {\hat{A}}_{k,2} & =\left[a_{z,k},a_{z,k}\right]\cdot diag\left({\left[\Gamma_{k}\right]}_{2,:}\right). \end{array}$$

The above equations indicate that ${\hat{A}}_{k,m}$ is a rank-1 matrix and has the same column space as the steering vector. Hence, the spacial frequency can be estimated by subspace methods. Giving the SVD of ${\hat{A}}_{k,m}$,

(29) $${\hat{A}}_{k,m}=U_{k,m}\Sigma_{k,m}V_{k,m}^{H},\;m=1,2,$$

the left singular vector $u_{k,1}$ corresponding to the maximum singular value can be obtained. Take the first and last $\left(M-1\right)$ rows of $u_{k,1}$, denoted as $u_{a}$ and $u_{b}$, to construct $U_{ab}=\left[u_{a},u_{b}\right]$. Applying the eigenvalue decomposition of $U_{ab}^{H}U_{ab}$,

(30) $$U_{ab}^{H}U_{ab}=W\Lambda W^{H},$$

in which:

(31) $$W=\left[\begin{array}{cc} w_{1,1} & w_{1,2}\\ w_{2,1} & w_{2,2} \end{array}\right]\in C^{2\times 2},$$

yields the estimation of spacial frequency ${\hat{\alpha }}_{k}=∠\left(-\frac{w_{1,2}}{w_{2,2}}\right)$, where $∠\left(\cdot \right)$ denotes the phase angle of a complex variable. Finally, the estimation of the arrival angle on the array along the *x*-axis can be obtained,

(32) $${\hat{\phi }}_{k}^{\prime }=\arccos\left(\frac{-{\hat{\alpha }}_{k}\lambda }{2\pi d}\right).$$

The same method can be used to get the estimation of the elevation angle ${\hat{\theta }}_{k}$. After obtaining the estimation of $\phi_{k}^{\prime }$ and $\theta_{k}$, the azimuth angle $\phi_{k}$ can be estimated directly,

(33) $${\hat{\phi }}_{k}=\arccos\left(\frac{\cos{\hat{\phi }}_{k}^{\prime }}{\sin{\hat{\theta }}_{k}}\right).$$

Since ${\hat{A}}_{k}$ includes the steering vector information on both subarrays and the Type-2 BCD is unique, the estimation of azimuth and elevation angles accomplish the matching pairing automatically. It should be noted that even if the array manifold is arbitrarily configured and no longer has the Vandermonde structure, the spatial frequency can still be estimated by utilizing the one-dimensional spectral peak search method.

The algorithm flow is shown in Algorithm 1. The ALS procedure is the key step to estimate the steering matrix, which has the complexity of $O\left(2K\left(P+1\right)\prod_{p=1}^{P}I_{p}\right)$. Such a procedure consists of three parts. The first is to update the factor matrices, which has the complexity of $O\left(2KP\prod_{p=1}^{P}I_{p}\right)$. The second is to solve the linear systems, which has the complexity of $O\left(\frac{1}{3}K^{3}P+2K^{2}\sum_{p=1}^{P}I_{p}\right)$. The third is function evaluation, which has the complexity of $O\left(2K\prod_{p=1}^{P}I_{p}\right)$. In addition, we employ the SVD to calculate the spatial frequencies, which has the the complexity of $O\left(2L_{1}^{3}\right)$. For the signal model of the EMVS array in this paper, we have P=3, $I_{1}=2M$, $I_{2}=6$, $I_{3}=N$. It can be concluded that the total computational complexity is $O\left(2K\left(3+1\right)\times \left(2M\times 6\times N\right)+2L_{1}^{3}\right)=O\left(96KMN+2L_{1}^{3}\right)$.
**Algorithm 1** 2D AOA estimation algorithm for the EMVS array based on Type-2 BCD.**Input:** the received signal $Y\in C^{2M\times 6\times N}$; the number of source signals, *K*; BCD model parameters, $L_{1}=L_{2}=2$; the threshold of error, ϵ.**Output:** AOA estimation ${\hat{\phi }}_{k},{\hat{\theta }}_{k},\;k=1,2,\ldots ,K$.  1: Initialization for factor matrices  2: $A=\left[A_{1},\ldots ,A_{K}\right],\;s.t.\;Col\left(A\right)=Col\left(Y_{2}Y_{1}^{†}\right)$;  3: $\Psi =\left[\Theta_{1},\ldots ,\Theta_{K}\right],\;s.t.\;Col\left(\Psi \right)=Col\left(Y_{2}^{T}{\left(Y_{1}^{T}\right)}^{†}\right)$;  4: Presetting for iteration variables  5: $\hat{S}=0_{L_{1}\times L_{2}\times N}$; $F=0_{6K\times L_{1}}$; $G=0_{2MK\times L_{2}}$; E=Y.  6: **while**
${∥E∥}_{F}>\epsilon$
**do**  7: ALS for estimating S: $\hat{S}\leftarrow Y_{\left(3\right)}{\left({\left[\hat{A}{⊙}_{b}\hat{\Psi }\right]}^{T}\right)}^{†}$;  8: ${\left[\hat{S_{k}}\right]}_{\left(3\right)}\leftarrow {\hat{S}}_{k}^{T},\;k=1,2,\ldots ,K$;  9: ${\hat{F}}_{k}\leftarrow {\left[{\hat{S}}_{k}\times_{2}{\hat{\Theta }}_{k}\right]}_{\left(1\right)},\;k=1,2,\ldots ,K$; 10: ALS for estimating A: $\tilde{A}\leftarrow Y_{\left(1\right)}{\left({\left[{\hat{F}}_{1}^{T},{\hat{F}}_{2}^{T},\ldots ,{\hat{F}}_{K}^{T}\right]}^{T}\right)}^{†}$; 11:  Apply QR decomposition: ${\tilde{A}}_{k}=Q_{A}R_{A}$, ${\hat{A}}_{k}\leftarrow Q_{A},\;k=1,2,\ldots ,K$; 12: ${\hat{G}}_{k}\leftarrow {\left[\hat{S_{k}}\times_{1}{\hat{A}}_{k}\right]}_{\left(2\right)},\;k=1,2,\ldots ,K$; 13: ALS for estimating Ψ: $\tilde{\Psi }\leftarrow Y_{\left(2\right)}{\left({\left[{\hat{G}}_{1}^{T},{\hat{G}}_{2}^{T},\ldots ,{\hat{G}}_{K}^{T}\right]}^{T}\right)}^{†}$; 14: Apply QR decomposition: ${\tilde{\Theta }}_{k}=Q_{\Theta }R_{\Theta }$, ${\hat{\Theta }}_{k}\leftarrow Q_{\Theta },\;k=1,2,\ldots ,K$; 15: Calculate the residual: $E\leftarrow Y-\sum_{k=1}^{K}{\hat{S}}_{k}\times_{1}{\hat{A}}_{k}\times_{2}{\hat{\Theta }}_{k}$; 16: **end while** 17: **while**
k≤K
**do** 18: Divide ${\hat{A}}_{k}$ into two parts: ${\hat{A}}_{k}={\left[{\hat{A}}_{k,1}^{T},{\hat{A}}_{k,2}^{T}\right]}^{T}$; 19: Apply SVD to each part: ${\hat{A}}_{k,m}=U_{k,m}\Sigma_{k,m}V_{k,m}^{H},\;m=1,2$; 20: Obtain the estimates ${\hat{\phi }}_{k}^{\prime },{\hat{\theta }}_{k}$ by the subspace approach. 21: ${\hat{\phi }}_{k}\leftarrow \arccos\left(\cos{\hat{\phi }}_{k}^{\prime }/\sin{\hat{\theta }}_{k}\right)$; 22: **end while**

## 4. Numerical Simulations and Discussion

In this section, several simulation experiments are demonstrated for proving the validation of the proposed method. The experimental scenario is consistent with the received signal model (14) described in Section 3.1. It is assumed that the L-shaped array consists of two ULAs, and each one has *M* sensors. The number of source signals is *K*; the number of snapshots is *N*; the Monte Carlo independent trials is *L*.

Since the signal studied in this paper is partially-polarized waves and the polarization state is time-varying, so the simulations in this section only estimate the AOA of the signal. In the numerical experiments, the polarization orientation angle is set as a random variable obeying the zero mean Gaussian distribution; the simulation results are given in the following subsections. If the distribution function changes to obeying the uniform distribution, the corresponding simulation results would have considerable consistency with the Gaussian distribution.

### 4.1. Implementations of 2D AOA Estimation

#### 4.1.1. Different SNRs

**Figure 5:**
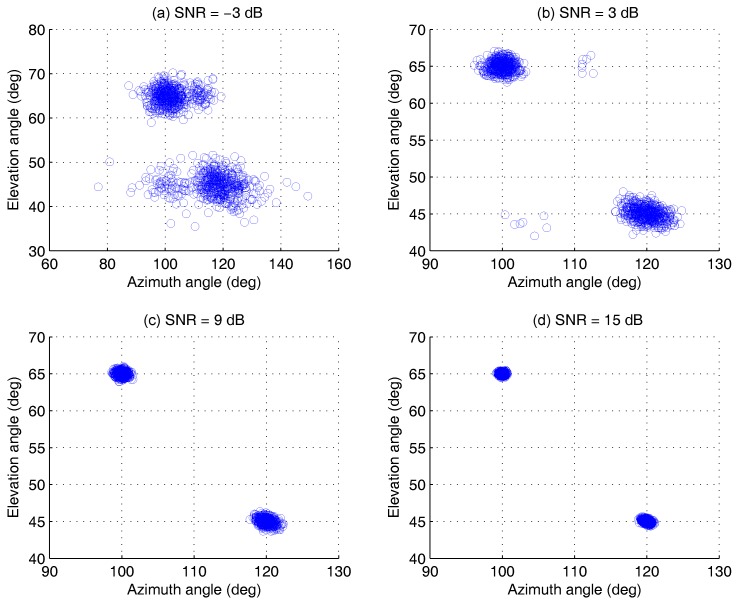
Azimuth-elevation scatter versus SNRs, for two AOAs at $\left(\phi_{1},\theta_{1}\right)=\left(100^{\circ },65^{\circ }\right)$, $\left(\phi_{2},\theta_{2}\right)=\left(120^{\circ },45^{\circ }\right)$, by 500 Monte Carlo trials (M=5,K=2,N=200). (**a**) SNR = –3 dB, (**b**) SNR = 3 dB, (**c**) SNR = 9 dB, (**d**) SNR = 15 dB.

The simulation parameters are configured as: M=5, K=2, N=200; the distance between adjacent sensors is λ/2; two groups of the AOAs are $\left(\phi_{1},\theta_{1}\right)=\left(100^{\circ },65^{\circ }\right)$ and $\left(\phi_{2},\theta_{2}\right)=\left(120^{\circ },45^{\circ }\right)$; the SNR range is −3 dB∼15 dB; the Monte Carlo trials L=500. The azimuth-elevation angles estimation is shown in Figure 5.

**Figure 6:**
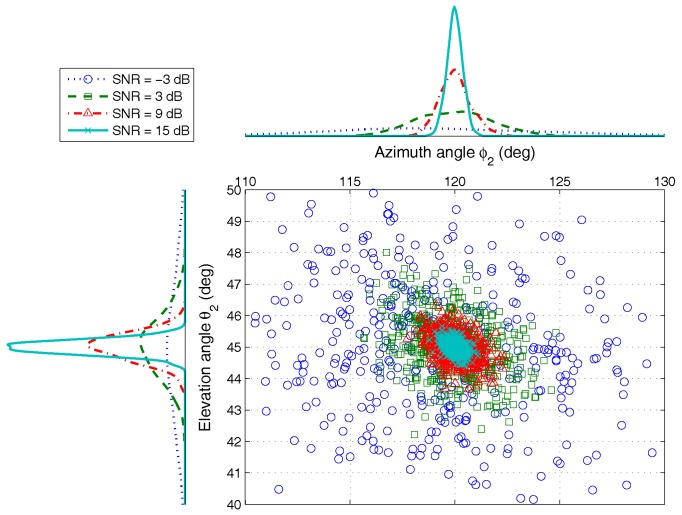
The AOA estimation scatter for $\left(\phi_{2},\theta_{2}\right)$ versus SNRs, by 500 Monte Carlo trials (M=5,K=2,N=200).

For comparing the accuracy of estimation under different SNRs more directly, we analyze the simulation result for one group of AOA, $\left(\phi_{2},\theta_{2}\right)$, as shown in Figure 6. The result indicates that the proposed algorithm has better estimation accuracy under different noise conditions. Even if the SNR is low, different AOAs can be distinguished, which can be verified by the result of detection probability in Section 4.2.2. When the SNR is −3 dB, the detection probability of the proposed algorithm is larger than 85%. However, the value of the existing method, e.g., ESPRIT, is less than 50% only.

#### 4.1.2. Different Angular Separations

**Figure 7:**
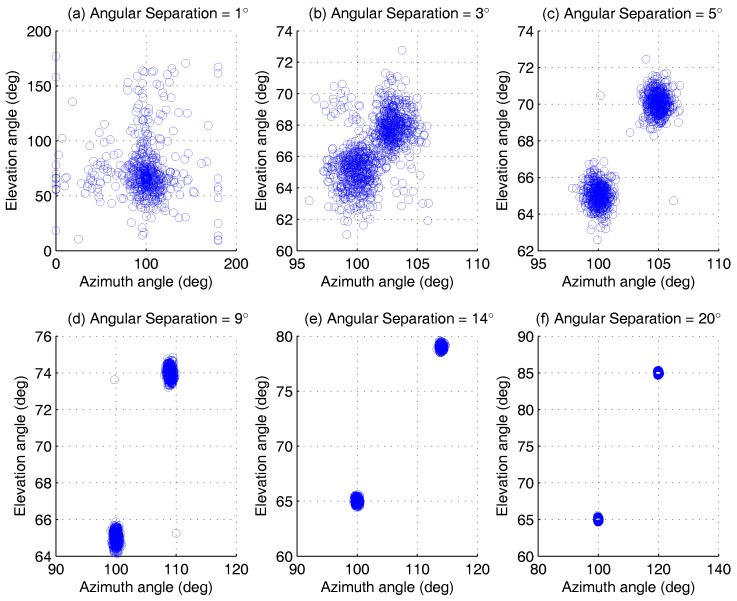
Azimuth-elevation scatter versus angular separations, for two AOAs at $\left(\phi_{1},\theta_{1}\right)=\left(100^{\circ },65^{\circ }\right)$, $\left(\phi_{2},\theta_{2}\right)=\left(100^{\circ }+\Delta ,65^{\circ }+\Delta \right)$, by 500 Monte Carlo trials (M=5,K=2,N=200). (**a**) Angular Separatio = $1^{\circ }$, (**b**) Angular Separatio = $3^{\circ }$, (**c**) Angular Separatio = $5^{\circ }$, (**d**) Angular Separatio = $9^{\circ }$, (**e**) Angular Separatio = $14^{\circ }$, (**f**) Angular Separatio = $20^{\circ }$.

This group of experiments investigates the estimation of AOA under different angular separations for fixed SNR, which is shown in Figure 7. Set SNR as 5 dB, and the remaining parameters agree with those of Section 4.1.1. Two groups of AOA are selected as $\left(\phi_{1},\theta_{1}\right)=\left(100^{\circ },65^{\circ }\right)$, $\left(\phi_{2},\theta_{2}\right)=\left(\phi_{1}+\Delta ,\theta_{1}+\Delta \right)$, $\Delta \in \left[1^{\circ },20^{\circ }\right]$. The Monte Carlo simulation trials L=500.

**Figure 8:**
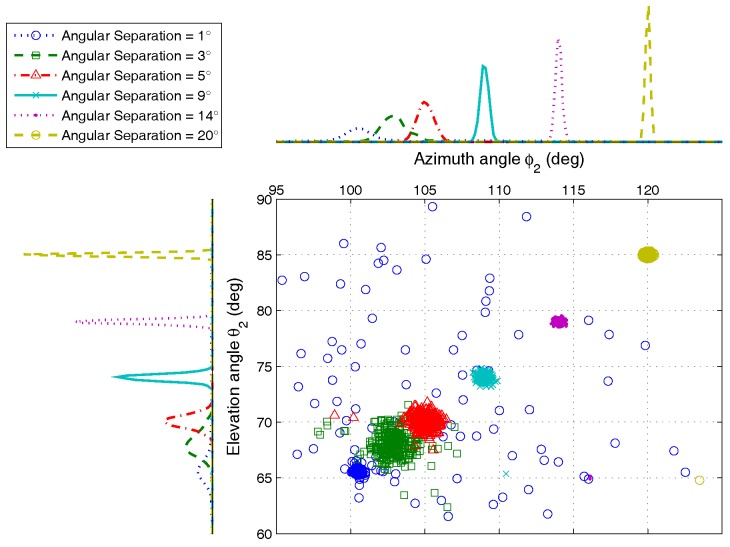
The AOA estimation scatter for $\left(\phi_{2},\theta_{2}\right)$ versus angular separations, by 500 Monte Carlo trials (M=5,K=2,N=200).

From the scatter diagram, the AOA estimation can be fully resolved when the angular separation is large than $3^{\circ }$. Even if the separation degree is very small, $\left(\phi_{2},\theta_{2}\right)$ can still be estimated correctly with high probability, as shown in Figure 8. This conclusion can also be proven by the result of detection probability analysis in Section 4.2.2. When $\Delta =1^{\circ }$, the detection probability of the proposed algorithm is larger than 80%. However, the ESPRIT has only about 40%.

#### 4.1.3. Different Snapshots

**Figure 9:**
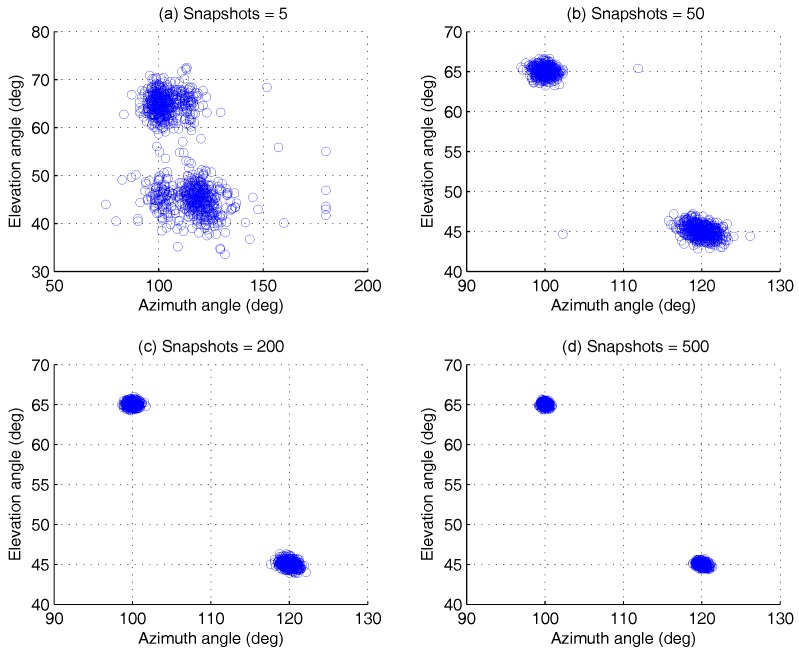
Azimuth-elevation scatter versus snapshots, for two AOAs at $\left(\phi_{1},\theta_{1}\right)=\left(100^{\circ },65^{\circ }\right)$, $\left(\phi_{2},\theta_{2}\right)=\left(120^{\circ },45^{\circ }\right)$, by 500 Monte Carlo trials (M=5,K=2). (**a**) snapshots = 5, (**b**) snapshots = 50, (**c**) snapshots = 200, (**d**) snapshots = 500.

This group of experiments investigates the estimation of AOA under different snapshots, which is set as $N\in \left[5,500\right]$. The SNR is fixed at 5 dB, and the remaining simulation parameters are the same as given in Section 4.1.1. With the given Monte Carlo trials L=500, the simulation result is shown in Figure 9.

**Figure 10:**
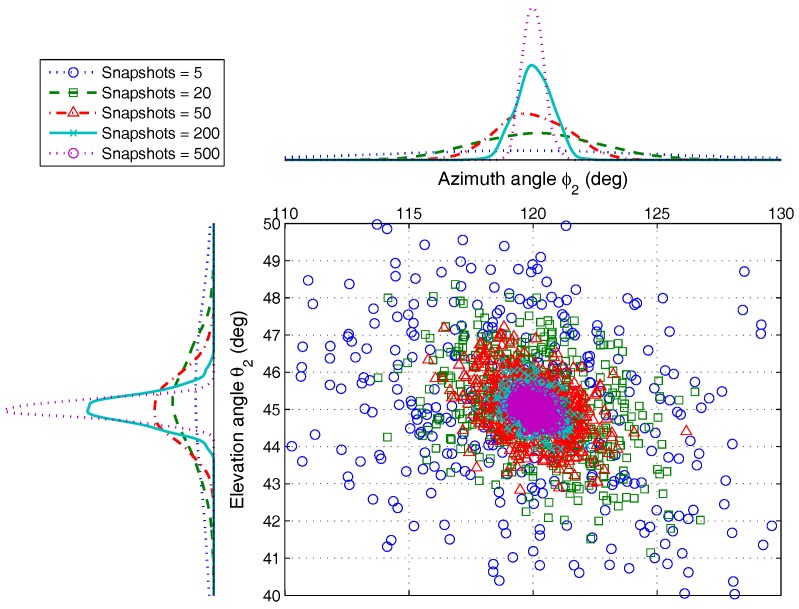
The AOA estimation scatter for $\left(\phi_{2},\theta_{2}\right)$ versus snapshots, by 500 Monte Carlo trials (M=5,K=2).

Even if the snapshots are finite, the simulation result shows that the proposed algorithm can achieve better angular resolution. For the different snapshots, the estimation of the two AOA groups appears to be hardly aliasing. When the snapshot is five, the detection probability with the proposed algorithm is more than 95% from the result in Section 4.2.2, while the detection probability of ESPRIT is less than 75%. One group of angle estimation results is shown in Figure 10. The simulation result reveals that the estimation accuracy of $\left(\phi_{2},\theta_{2}\right)$ rises with the snapshot increasing.

### 4.2. Performance Comparison

The comparison of the proposed algorithm with three existing algorithms has been investigated in this section, which shows the performance advantage of it in both root mean square error (RMSE) and detection probability. The reference methods include subspace-like algorithms, i.e., TM-MUSIC [27], ESPRIT [58,59], and the CPD-based 2D AOA estimation algorithm [26]. The ESPRIT algorithm is based on matrix modeling, the TM-MUSIC and CPD algorithms are based on tensor modeling.

#### 4.2.1. RMSE

First, we investigate the RMSE of each method, which is defined as:

(34) $$\mathrm{RMSE}=\sqrt{\frac{1}{LK}\sum_{l=1}^{L}\sum_{k=1}^{K}\left({\left({\hat{\phi }}_{k}\left(l\right)-\phi_{k}\right)}^{2}+{\left({\hat{\theta }}_{k}\left(l\right)-\theta_{k}\right)}^{2}\right)},$$

where $\left({\hat{\phi }}_{k}\left(l\right),{\hat{\theta }}_{k}\left(l\right)\right)$ are the estimation values of the *k*-th AOA for the *l*-th Monte Carlo trial. The Cramer–Rao lower bound is defined as $\sqrt{\frac{1}{K}\sum_{k=1}^{K}\left(CRB\left(\phi_{k}\right)+CRB\left(\theta_{k}\right)\right)}$, where $CRB\left(\phi_{k}\right)$ and $CRB\left(\theta_{k}\right)$ are the diagonal elements of CRB matrix [60,61], corresponding to the parameters $\phi_{k}$ and $\theta_{k}$, respectively.

**Figure 11:**
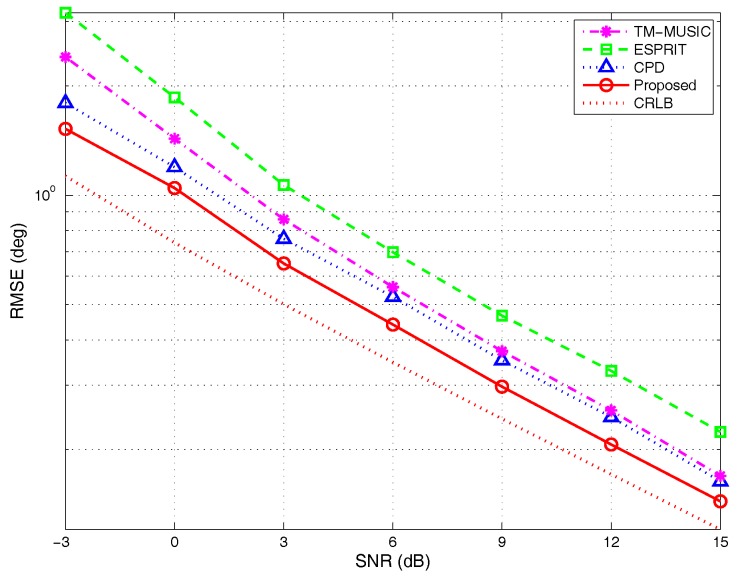
RMSE versus SNRs, by 500 Monte Carlo trials (M=5,K=2,N=200).

(1) RMSE versus different SNRs:

The parameter settings are the same as those in the Section 4.1.1. The results are shown in Figure 11. The RMSE curves interpret the advantage of the algorithm developed in this paper over the reference methods in the whole SNR range. The three tensor-based algorithms are better than the matrix-based algorithm, i.e., ESPRIT, since tensor modeling can make the best of the multidimensional structure information in the received signal. It is noticed that when the SNR is high, e.g., 15 dB, the performance of TM-MUSIC is close to the CPD algorithm. Compared with the CPD algorithm, as analyzed in Section 3.2, the BCD algorithm can solve the steering matrix $A_{k}$ directly and then automatically achieve the matching pair of the azimuth and elevation angles. Thus, the detection probability of BCD is higher than CPD, which utilizes the pair-matching technique based on the cross-correlation matrix (CCM) [26]. The results in Section 4.2.2 provide the same conclusion. Therefore, the performance of the proposed algorithm is better than that of the CPD algorithm. As a classic matrix subspace algorithm, the RMSE of the ESPRIT algorithm is the highest. This may be because ESPRIT has to lose a part of array aperture for constructing the rotation-invariant matrix.

**Figure 12:**
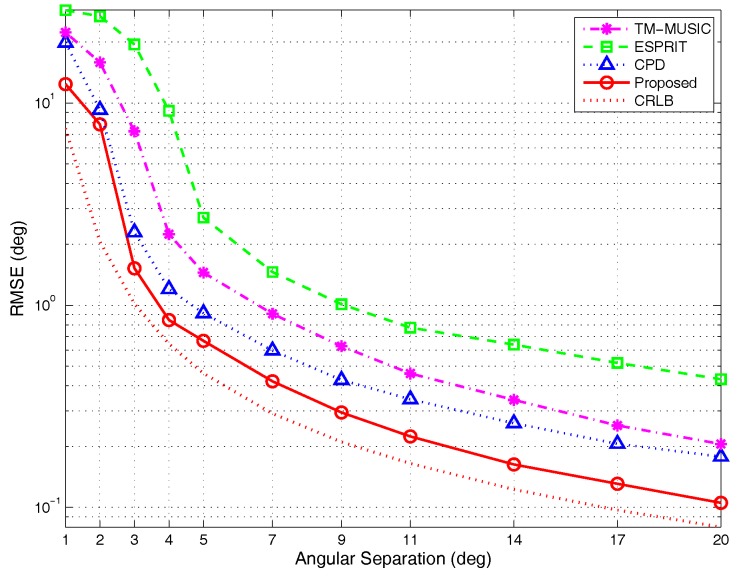
RMSE versus angular separations, by 500 Monte Carlo trials (M=5,K=2,N=200).

(2) RMSE versus different angular separations:

The parameter settings are taken as those in the Section 4.1.2. Figure 12 shows the simulation results under different angular separations. The RMSE curves vary with the steeper slope as the separation degree is less than $5^{\circ }$, which reveals the sensitivity to angular separation within such an interval. The results of detection probability analysis in Section 4.2.2 can also verify this conclusion. The curves in Figure 15 indicate that the detection probability is larger than 95% for all algorithms with the angular separation larger than $5^{\circ }$. Below this value, the detection probability is diverse for different algorithms. It is noticed that when the angular separation is $1^{\circ }$, of the three reference methods, the performance of TM-MUSIC algorithm approaches the CPD. When the angular separation is increasing, the RMSE of TM-MUSIC is becoming larger than the tensor-decomposition-based algorithms and so is the ESPRIT algorithm. Essentially, the TM-MUSIC is a kind of smoothing approach along a certain mode. Therefore, it can achieve a better performance than the classic subspace method, which may be influenced by outliers when the parameters to be estimated are close. Of all of the methods, the proposed algorithm has the lowest RMSE, which shows its high-resolution feature under the condition of small angular separation.

**Figure 13:**
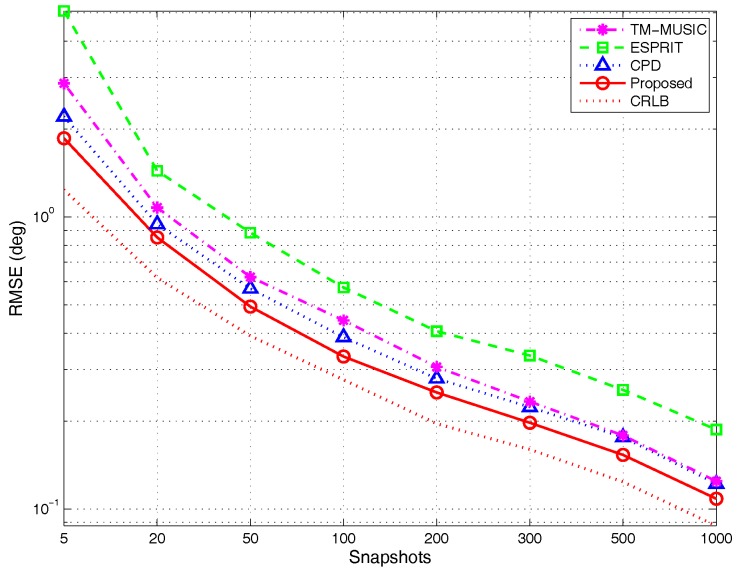
RMSE versus snapshots, by 500 Monte Carlo trials (M=5,K=2).

(3) RMSE versus different snapshots:

The experimental parameters are set as those in the Section 4.1.3. The simulation results are depicted in Figure 13. The RMSE curves indicate that the tensor-based methods outperform the classic matrix-based subspace method ESPRIT during the entire snapshots’ range. The RMSE of TM-MUSIC approximates that of CPD algorithm when the snapshots are larger than 300. The analysis in Section 3.1.1 indicates that when K=2, the uniqueness of BCD can be met as long as snapshots N>3. The similar conclusion is also suitable for the CPD algorithm. The results of detection probability analysis in Section 4.2.2 also provide the same conclusion that even for limited snapshots, BCD modeling can keep the detection probability on a higher level. Consequently, the performance of the proposed algorithm is better than the reference algorithms.

#### 4.2.2. Detection Probability

The detection probability measures the success rate of pair-matching for two group of azimuth-elevation angles. In this section, detection probabilities of the four methods are compared in accordance with three simulation experiments, grouped by SNR, angular separation and snapshot.

**Figure 14:**
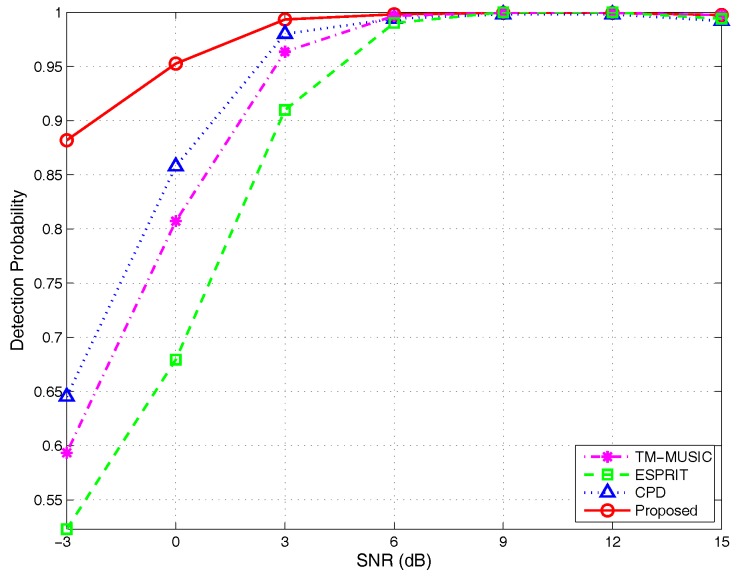
Detection probability versus SNRs, by 500 Monte Carlo trials (M=5,K=2,N=200).

(1) Detection probability versus different SNRs:

The experiment parameters as set in the Section 4.1.1 yield the simulation results shown in Figure 14. The detection probability curves show the obvious advantage of the proposed algorithm in the success rate of angle pair-matching at low SNR. For example, when SNR = −3 dB, the detection probability of the proposed algorithm reaches 88% and increased by 69.2%, 49.1% and 35.4% compared with ESPRIT (52%), TM-MUSIC (59%) and CPD (65%), respectively. This benefit by automatic angle pairing resulted from estimating steering vectors and the decomposition uniqueness of BCD, which makes the proposed algorithm obtain more powerful detection ability and possess the best estimation performance under the worse noise environment.

**Figure 15:**
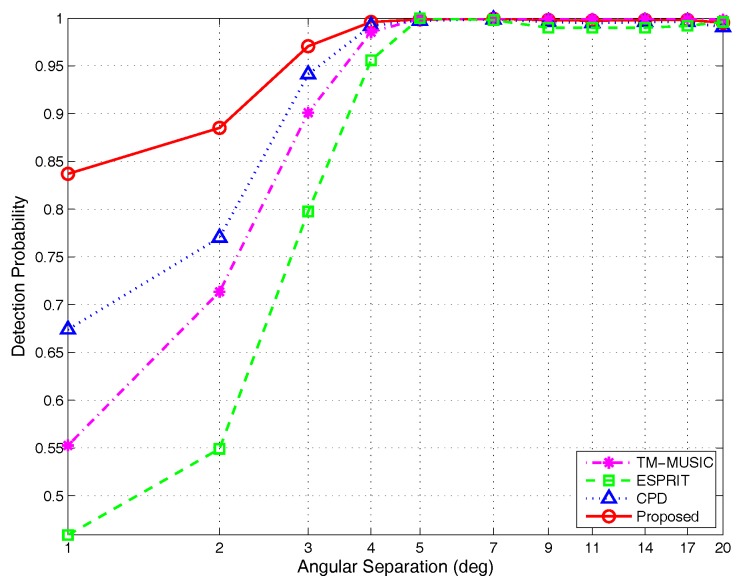
Detection probability versus angular separations, by 500 Monte Carlo trials (M=5,K=2,N=200).

(2) Detection probability versus different angular separations:

The experimental parameters are the same as those in Section 4.1.2. Figure 15 shows the simulation results. We can find that for different angular separations, the algorithm developed in this paper always has the highest success rate of angle pair-matching, especially at a small angle separation degree ($\Delta \leq 3^{\circ }$). For instance, when $\Delta =1^{\circ }$, the success rate of the proposed algorithm is 84% and increased by about 82.6%, 52.7% and 25.4% compared with ESPRIT (46%), TM-MUSIC (55%) and CPD (67%), respectively. When $\Delta \in (3^{\circ },5^{\circ })$, the detection probabilities of all methods have significant improvement. Meanwhile, the proposed algorithm is still optimal. When $\Delta >5^{\circ }$, all of the methods achieve 100% detection probability.

**Figure 16:**
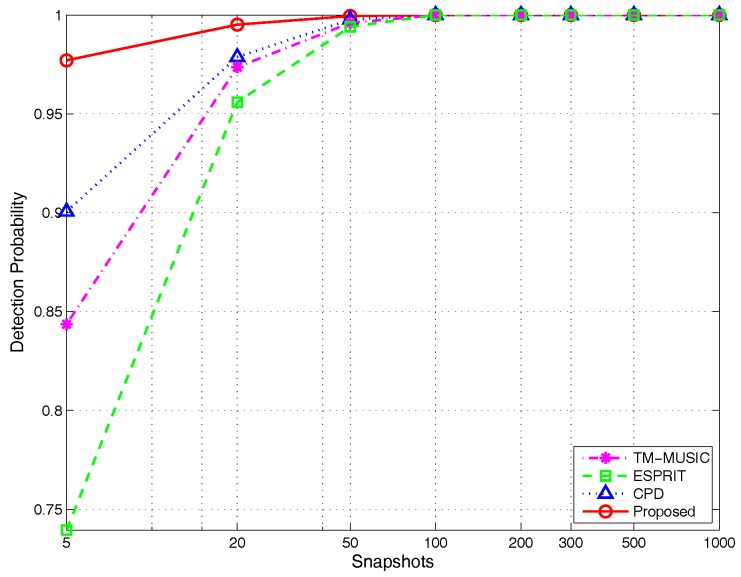
Detection probability versus snapshots, by 500 Monte Carlo trials (M=5,K=2).

(3) Detection probability versus different snapshots:

The experimental parameters are selected as set in Section 4.1.3. The simulation results can be seen in Figure 16. The detection probability curves show that the proposed algorithm has a higher success rate of angle pair-matching versus different snapshots, especially at small snapshots. As an example, when the snapshot is five, the detection probability of the proposed algorithm reaches to 97% and increased by about 31.1%, 15.4% and 7.8% compared with ESPRIT (74%), TM-MUSIC (84%) and CPD (90%), respectively. What is more, one more interesting characteristic different from the reference methods is that the detection probability of the proposed algorithm is slightly influenced by snapshots, which is embodied by the evenly-varied curve. This characteristic makes the proposed algorithm possess the highest detection probability even under the limited snapshots. Hence, the algorithm is more suitable for the scenario with the real-time requirement.

## 5. Conclusions

The application of BCD tensor modeling in parameter estimation of the EMVS array has been studied in this paper. In allusion to the issue of lacking effective methods for solving parameter estimation of partially-polarized waves, we put forward an algorithm for 2D AOA estimation of the EMVS array based on the rank-$\left(L_{1},L_{2},\cdot \right)$ BCD model. This algorithm can make full use of the multiway structural information of the received signal and achieve a high-resolution estimation. Benefiting from the decomposition uniqueness of such a Type-2 BCD model, the pair-matching of azimuth and elevation angles can be automatically achieved. According to the possible situations in practical applications, we carry out several groups of numerical experiments to verify the effectiveness of the proposed algorithm. The experimental results show that both the estimation accuracy and detection success rate of this algorithm are superior to the existing subspace method based on matrix decomposition and the CPD method based on tensor decomposition. The proposed algorithm manifests robust and good performance under severe conditions, such as low SNR, small angular separation and limited snapshots. This makes the algorithm possess potential application value in practical engineering applications.
